# Genome-Wide Characterization of the MADS-Box Gene Family in Radish (*Raphanus sativus* L.) and Assessment of Its Roles in Flowering and Floral Organogenesis

**DOI:** 10.3389/fpls.2016.01390

**Published:** 2016-09-20

**Authors:** Chao Li, Yan Wang, Liang Xu, Shanshan Nie, Yinglong Chen, Dongyi Liang, Xiaochuan Sun, Benard K. Karanja, Xiaobo Luo, Liwang Liu

**Affiliations:** ^1^National Key Laboratory of Crop Genetics and Germplasm Enhancement, College of Horticulture, Nanjing Agricultural UniversityNanjing, China; ^2^School of Earth and Environment, The UWA Institute of Agriculture, The University of Western AustraliaPerth, WA, Australia

**Keywords:** radish, MADS-box genes, flowering, floral organogenesis, RT-qPCR

## Abstract

The MADS-box gene family is an important transcription factor (TF) family that is involved in various aspects of plant growth and development, especially flowering time and floral organogenesis. Although it has been reported in many plant species, the systematic identification and characterization of MADS-box TF family is still limited in radish (*Raphanus sativus* L.). In the present study, a comprehensive analysis of MADS-box genes was performed, and a total of 144 MADS-box family members were identified from the whole radish genome. Meanwhile, a detailed list of MADS-box genes from other 28 plant species was also investigated. Through the phylogenetic analysis between radish and *Arabidopsis thaliana*, all the *RsMADS* genes were classified into two groups including 68 type I (31 Mα, 12 Mβ and 25Mγ) and 76 type II (70 MIKC^C^ and 6 MIKC^∗^). Among them, 41 (28.47%) *RsMADS* genes were located in nine linkage groups of radish from R1 to R9. Moreover, the homologous MADS-box gene pairs were identified among radish, *A. thaliana*, Chinese cabbage and rice. Additionally, the expression profiles of *RsMADS* genes were systematically investigated in different tissues and growth stages. Furthermore, quantitative real-time PCR analysis was employed to validate expression patterns of some crucial *RsMADS* genes. These results could provide a valuable resource to explore the potential functions of *RsMADS* genes in radish, and facilitate dissecting MADS-box gene-mediated molecular mechanisms underlying flowering and floral organogenesis in root vegetable crops.

## Introduction

MADS-box genes, which were primarily identified as floral homeotic genes, encode a family of transcription factors (TFs) containing a highly conserved MADS domain of approximately 60-amino-acid sequences in the N-terminal region (Norman et al., 1988), which bind to (CC[A/T]_6_GG) that is also known as CArG boxes (Pellegrini et al., 1995; Shore and Sharrocks, 1995; Sasaki et al., 2010). Based on phylogenetic relationships, MADS-box genes have been classified into two broad groups, type I and type II genes, which were generated by single gene duplication (Alvarez-Buylla et al., 2000; Liu et al., 2013). Among them, type I proteins are further divided into three subgroups including Mα, Mβ and Mγ, while type II can be classified into the subgroups MIKC^C^ and MIKC^∗^ according to the sequence divergence at I domain (De Bodt et al., 2003; Wells et al., 2015). The MIKC^∗^ type proteins have a longer I domain and a less conserved K domain than the MIKC^C^ type (Henschel et al., 2002; Gramzow and Theißen, 2013). Previous reports revealed the type I MADS-box genes encode SRF-like domain proteins, while type II genes encode MEF2-like proteins and MIKC-type proteins (De Bodt et al., 2003; Wells et al., 2015). Intriguingly, the most well-known MADS-box proteins belong to MIKC-type proteins which contains four common domains including MADS (M), Intervening (I), Keratin (K) and the C-terminal (C) domain (Kaufmann et al., 2005). Compared with type II, the type I proteins lack the K domain and show a relatively simple gene structure that usually only have one or two exons (Smaczniak et al., 2012; Kaufmann et al., 2005). At present, 62 Type I and 46 Type II genes have been identified and characterized in *A. thaliana* (Parenicová et al., 2003). Among the 46 Type II genes, 39 MIKC^C^ type genes were further classified into 12 groups based on their phylogenetic relationships, nevertheless, there were only seven genes belonging to the MIKC^∗^ type (Duan et al., 2015).

In plants, increasing evidences from genetic and molecular analyses have revealed that MADS-box genes could play critical roles in regulating diverse developmental processes, such as flower organogenesis (Zahn et al., 2006), determination of flowering time (Moon et al., 2003; Adamczyk et al., 2007; Lee et al., 2007; Liu et al., 2008; Hu et al., 2014), regulation of fruit ripening (Liljegren et al., 2000), development of vegetative organs (Tapia-López et al., 2008), seed pigmentation and embryo development (Nesi et al., 2002). MIKC^C^-type MADS-box genes play fundamental roles especially in flowering time control and floral organ identity. Based on the proposed ABC model (Haughn and Somerville, 1988), the ABCDE model that determines the identity of floral organs has been presented. Different floral organs identities are controlled by various combinations of types of genes, sepals (A+E), petals (A+B+E), stamens (B+C+E), carpels (C+E) and ovules (D+E) (Zahn et al., 2006). A series of correlative functional genes were found to be involved in this process, such as Class A, *APETALA1* (*AP1*); Class B, *PISTILATA* (*PI*) and *AP3*; Class C, *AGAMOUS* (*AG*); Class D, *SEEDSTICK* (*STK*); and Class E, *SEPALLATA* (*SEP1*, *SEP2*, *SEP3* and *SEP4*) (Parenicová et al., 2003).

In recent decades, several crucial MIKC^C^-type genes have been suggested to modulate flowering time in *A. thaliana.* For instance, *FLOWERING LOCUS C* (*FLC*) gene has been found to inhibit flowering by encoding a specific MADS domain protein (Michaels and Amasino, 1999). *SUPPRESSOR OF OVEREXPRESSION OF CO1* (*SOC1*) gene plays a critical role in vernalization and gibberellin signal integration for flowering (Moon et al., 2003). *SHORT VEGETATIVE PHASE* (*SVP*) is considered as an important control factor of flowering time by ambient temperature (Lee et al., 2007). Moreover, *AGAMOUS-LIKE16 (AGL16)* gene targeted by miR824 contributes to the repression of plant flowering time (Hu et al., 2014).In addition, several other MIKC^C^-type genes have also been proven to be involved in flowering time, such as *AGAMOUS-LIKE 24* (*AGL24*) (Liu et al., 2008), *MADS AFFECTING FLOWERING* (*MAF1/FLM*) (Ratcliffe et al., 2003) and *AGAMOUS-LIKE 15/18 (AGL15*/*AGL18*) (Adamczyk et al., 2007). More intriguingly, compared to MIKC^C^-type genes, relatively less study has been conducted on the functions of MIKC^∗^-type and Type I genes. To date, MIKC^∗^-type and Type I genes only have been shown to participate in the *A. thaliana* male and female gametophyte, respectively (Zobell et al., 2010; Masiero et al., 2011). Furthermore, recent studies have revealed that Type I genes are primarily expressed in developing seed of *A. thaliana* (Barker and Ashton, 2013).

Radish (*Raphanus sativus* L., 2*n* = 2*x* = 18) is an important root vegetable crop of Brassicaceae family worldwide (Xu et al., 2013). In the complete life cycle of radish, bolting and flowering are some of the critical factors which affect the yield and quality. Premature bolting seriously decreases the production of vegetable crops which ultimately lead to the reduction of economic benefits (Nie et al., 2015). Consequently, it is extremely essential to explore the MADS-box gene family whose primary function is to regulate flowering time and floral organ development. Recently, genome-wide identification and characterization of MADS-box genes were reported in some plant species including *A. thaliana* (Parenicová et al., 2003), rice (Arora et al., 2007), Chinese cabbage (Duan et al., 2015), cucumber (Hu and Liu, 2012), soybean (Fan et al., 2013) and maize (Zhao et al., 2011). However, the genome-wide analysis and characterization of MADS-box genes in radish remain lacking. Especially, it is ambiguous how MADS-box genes control flowering time and floral organ development in radish. Fortunately, the completion of the radish genome sequencing makes it possible to analyze MADS-box genes (Mitsui et al., 2015). In the present study, MADS-box members from radish genome were firstly identified and divided into different classes, and the gene structures, conserved motifs and phylogenetic relationships between these members were systematically analyzed. Additionally, linkage group locations and primary prediction of gene functions were also investigated, and the expression patterns of all MIKC^C^ genes in radish were carried out with RT-qPCR. These results would greatly contribute to gain insight into functional analysis of MADS-box genes and facilitate dissecting MADS-box gene-mediated molecular mechanisms underlying flowering and floral organogenesis in radish and other root vegetable crops.

## Materials and Methods

### Identification of MADS-Box Genes

All radish genome sequences used to identify the MADS-box genes were available from the NODAI Radish genome database^1^ (Mitsui et al., 2015). To confirm the candidates of radish MADS-box genes, the proteins with SRF-TF domain (Pfam accession number:PF00319)^2^ were searched against the genome protein sequences using HMM search tool with an *E*-value cut-off 1.0 (Finn et al., 2011; Finn et al., 2015). Each sequence predicted was subsequently verified through the public databases including NCBI^3^, Pfam and SMART^4^ to confirm its reliability (Letunic et al., 2012).

### Sequence Collection from Various Plant Species

The MADS-box protein sequences of *A. thaliana* and Chinese cabbage were downloaded from TAIR database^5^ and *Brassica* database (BRAD^6^) (Wang et al., 2011), respectively. *Capsicum annuum* and *Brassica oleracea* genome protein sequences were retrieved from pepper genome platform^7^ (Kim et al., 2014) and *Brassica* database, respectively. The genome data of *Beta vulgaris*, *Fragaria vesca*, *Phaseolus vulgaris*, *Ricinus communis*, *Brachypodium distachyon*, *Setaria italica*, *Amborella trichopoda* and *Chlamydomonas reinhardtii* were downloaded from the genome browser phytozome^8^. All these collected genome sequences were used to screen MADS-box genes from various plant species through the Pfam database. All the sequences of the other species used in this study were collected from previous reports (Parenicová et al., 2003; Leseberg et al., 2006; Arora et al., 2007; Díaz-Riquelme et al., 2009; Zhao et al., 2011; Gramzow et al., 2012; Barker and Ashton, 2013; Fan et al., 2013; Duan et al., 2015).

### Linkage Group Localization and Identification of Orthologous and Paralogous Genes

The sequences of *RsMADS* genes were searched against the genomic sequences of the scaffolds that were anchored to the integrated high-density linkage map (Kitashiba et al., 2014). The gene sequences with identity ≥98% and length difference ≤5 bp were considered to be the same genes between the two genomes, and localized to the linkage groups according to the corresponding location parameters using MapInspect Software^9^.

To gain insight into the homology relationship between MADS-box genes of radish and other species, we investigated the orthologous and paralogous MADS-box genes in radish, *A. thaliana*, Chinese cabbage and rice using OrthoMCL program^10^ (Li et al., 2003). Subsequently, the relationship networks of homologous genes in radish and *A. thaliana* was visualized using Cytoscape software (Shannon et al., 2003).

### Identification of Protein Properties, Gene Structure and Conserved Motifs and Phylogenetic Analysis

ProtParam tool of ExPASy^11^ was employed to analyze series of RsMADS protein properties like molecular weight, theoretical pI and instability index. The Pfam database and SMART were employed to determine conserved domains of proteins. After that, the GSDS^12^ was adopted to reveal intron-exon structure of *RsMADS* genes. Conserved motifs were identified using Motif Elicitation (MEME) software^13^, and the parameters settings as follows: (1) 10 ≤ optimum motif width ≤100; and (2) maximum number of motifs = 15. In addition, multiple alignments of MADS-box gene sequences were performed using ClustalX 2.0 with default parameters. MEGA 5.1 (Tamura et al., 2011) was then used to construct the phylogenetic tree based on neighbor-joining (NJ) method, and bootstrap values were set to 1,000 replications.

### Prediction of miRNAs Targeting the *RsMADS* Genes

To identify potential miRNAs targeting the *RsMADS* genes, all *RsMADS* genes were searched against a comprehensive miRNA library on psRNATarget Server^14^ with default parameters (Dai and Zhao, 2011), which was constructed according to the previously established five miRNA libraries (Xu et al., 2013; Nie et al., 2015; Sun X. et al., 2015; Sun Y. et al., 2015; Yu et al., 2015). After that, Cytoscape software was utilized to visualize the targeted relationship between predicted miRNA and corresponding *RsMADS* genes.

### Expression Analysis Using Radish RNA-seq Data

Illumina RNA sequencing showed gene expression of radish varied in the different tissues and developmental stages (Mitsui et al., 2015). In this study, the Illumina RNA-Seq data, which were downloaded from NODAI radish genome database, were used for the transcriptional profiling of *RsMADS* genes in five tissues (cortical, cambium, xylem, root tip and leaf) and six stages of leaf [7, 14, 20, 40, 60 and 90 days after sowing(DAS)]. The expression level for each *RsMADS* gene was presented by the RPKM (Reads Per kb per Million reads) method (Mitsui et al., 2015). Lastly, heat maps were generated by Cluster 3.0^15^ (de Hoon et al., 2004) and Tree View^16^ (Saldanha, 2004).

### Plant Material and Treatments

The radish advanced inbred line, ‘NAU-DY13,’ was used in the current study. Germinated seeds were vernalized and sown in plastic pots and cultivated in controlled-environment growth chamber with day/night temperature of 28/18°C. For vernalization treatments, germinated seeds were vernalized at 2–4°C for 0, 10 and 30 days, respectively, and grow under the middle-day (12 h light/12 h dark). For photoperiodic treatments, unvernalized seedlings were cultured under long-day (16 h light/8 h dark) and short-day (8 h light/16 h dark) treatments, respectively. Furthermore, the rest of unvernalized seedlings were treated with 200 mg/L and 800 mg/L GA_3_ every other day for a week from 2-week-old seedlings under the middle-day condition. Unvernalized seedlings without any treatment grown under the middle-day were set as control (CK). Leaf samples were collected when treated seedlings were grown to three weeks old. Different flower tissues from control plants, including sepal, petal, stamen and carpel, were collected separately at reproductive stage. All the samples were collected from three randomly selected individuals and immediately frozen in liquid nitrogen and stored at -80°C for further use.

### RNA Isolation and RT-qPCR

Total RNA of each sample was isolated using Trizol reagent according to the manufacturer’s instructions (Invitrogen, Carlsbad, CA, USA). Then, the first-strand cDNA was synthesized using the Superscript III First-Strand Synthesis System (Invitrogen). The specific primers of *RsMADS* genes for RT-qPCR were designed using Beacon Designer 7.7 (Premier Biosoft International, Palo Alto, CA, USA). To confirm results reliability, three biological and three technical replicates were adopted. RT-qPCR reaction system and cycling profile were carried out on Bio-Rad iQ5 Real-Time PCR System. *RsActin* gene was selected as the reference gene (Xu et al., 2013). The primers used for RT-qPCR were shown in **Supplementary Table S1**. Finally, formula -ΔΔ*C*T and 2^-ΔΔ^^*C*T^ were applied to calculate the relative expression ratio. The data were statistically analyzed with Duncan’s multiple range test at the *P* < 0.05 level of significance using SPSS 20 software (SPSS Inc., USA).

## Results

### Identification and Analysis of MADS-Box Proteins in Radish

**FIGURE 1:**
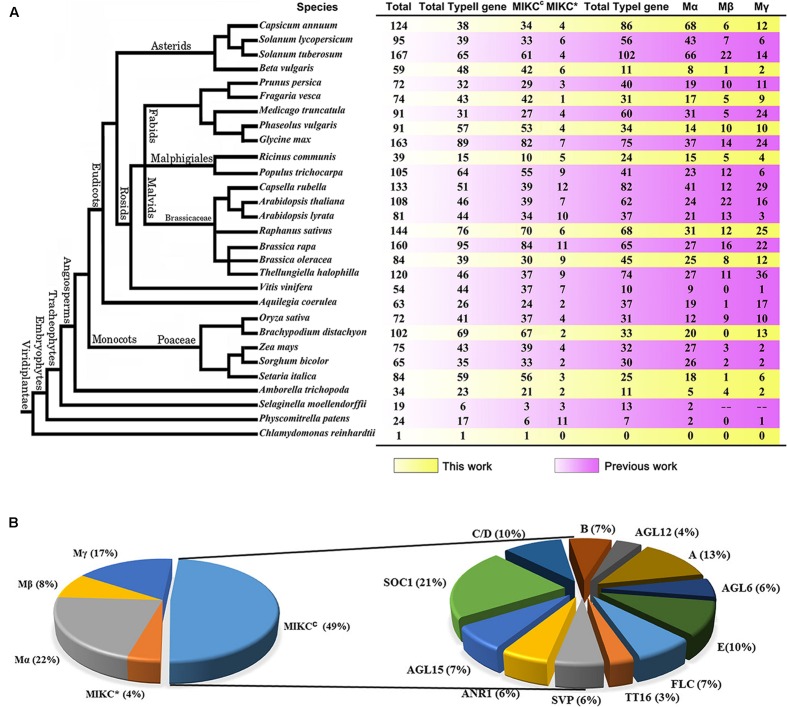
**The classification of MADS-box family genes. (A)** The number of the MADS-box family genes of 29 plant species. **(B)** The classification and proportions of *RsMADS* genes.

To define the candidate MADS-box proteins in radish, a profile hidden Markov model (HMM) search against NODAI radish genome protein sequences was carried out using the SRF-TF domain (PF00319), and totally 157 putative MADS-box protein genes were obtained. The low quality sequences without start and/or stop codons were removed to ensure the reliability of these sequences, and finally a total of 146 sequences were retained. Subsequently, all remaining sequences were verified through the public databases including NCBI, Pfam and SMART. All these radish MADS-box proteins were named as RsMADS001 to RsMADS146, respectively (**Supplementary Table S2**). After searching these protein sequences against *A. thaliana* on TAIR database by BLASTP, RsMADS040 and RsMADS091 were removed, because they contained other functional domains and their homologous proteins were non-MADS-box proteins (**Supplementary Figure S1**). To study the comparative evolution among various plant species, MADS-box genes from 28 other plant species were also collected by searching for SRF-TF domain (PF00319) in their genomes (**Figure 1A**; **Supplementary Tables S3** and **S4**). Compared with other species, radish had a relatively large MADS-box gene family of 144 members, and the members of MADS-box gene family subgroups were also identified (**Figure 1A**).

### Comparative Phylogenetic Analysis of *RsMADS* Genes

To better understand the phylogenetic relationships of the MADS-box genes between radish and *A. thaliana*, the classification of 108 MADS-box genes from *A. thaliana* was performed (**Supplementary Figure S2A**). An unrooted phylogenetic tree of MADS-box genes between radish and *A. thaliana* was constructed by the NJ method (**Supplementary Figure S2B**). It is quite obvious that *RsMADS* genes were divided into five clades according to the classification of the *A. thaliana*, namely subfamilies MIKC^C^ (70), MIKC^∗^ (6), Mα (31), Mβ (12) and Mγ (25) (**Supplementary Figures S2B,D**). Additionally, an unrooted phylogenetic tree was produced using MADS-box proteins from radish, *A. thaliana* and Chinese cabbage to further confirm the phylogenetic relationships and classification of RsMADS proteins (**Supplementary Figure S2C**).

**FIGURE 2:**
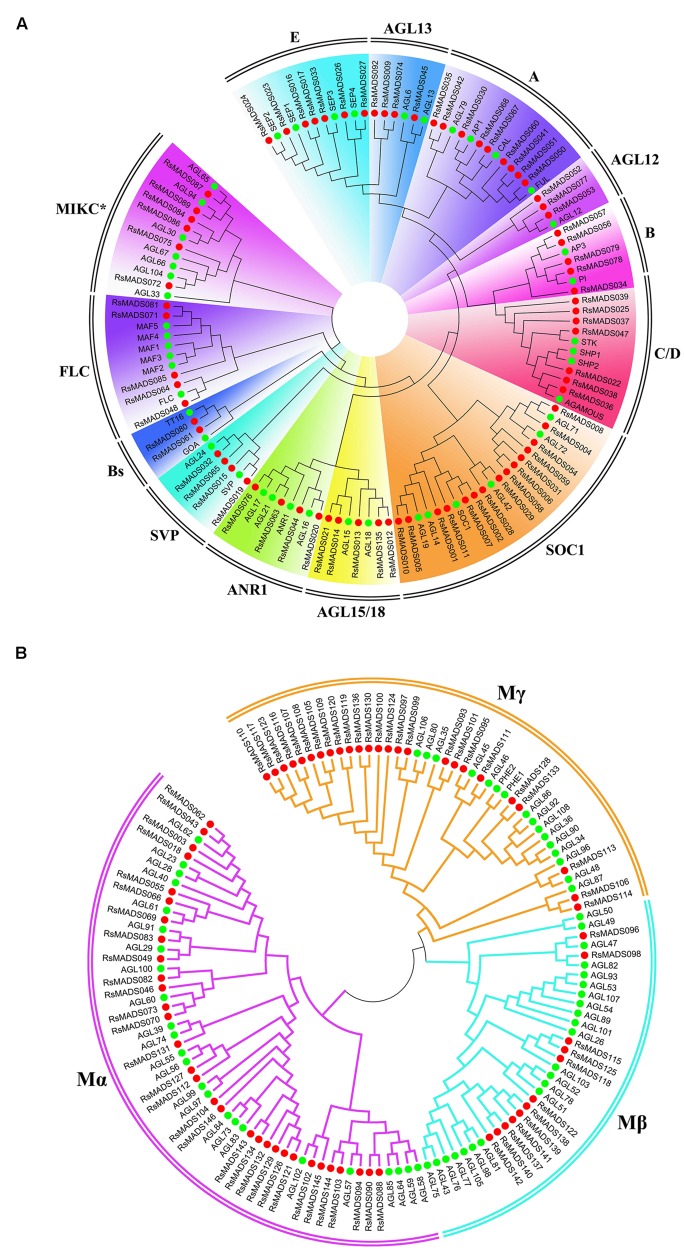
**Phylogenetic relationship of radish and *A. thaliana* type II **(A)** and type I **(B)** MADS-box proteins**. Subgroups are marked in different colors.

Phylogenetic trees for type I and type II MADS-box genes were separately generated using *A. thaliana* and radish proteins (**Figures 2A,B**). Totally 49% (70) of the 144 *RsMADS* genes belongs to the MIKC^C^-type genes, which could be further divided into 12 subfamilies (**Figures 1B and 2A**). Subgroup SOC1 and subgroup Bs, respectively, showed the largest (∼21%) and smallest (∼3%) number of *RsMADS* genes. However, in *A. thaliana* the largest and smallest proportion of subgroup is ∼15% (subgroup SOC1 or FLC) and ∼3% (subgroup AGL12), respectively (Parenicová et al., 2003). The number of *RsMADS* genes from other subgroups ranged from four to 13, interestingly, subgroup C/D and E had seven members, while subgroup B, FLC and AGL15 consisted of five members (**Figures 1B and 2A**).

### Linkage Group Localization and Orthologous Relationship Analysis

**FIGURE 3:**
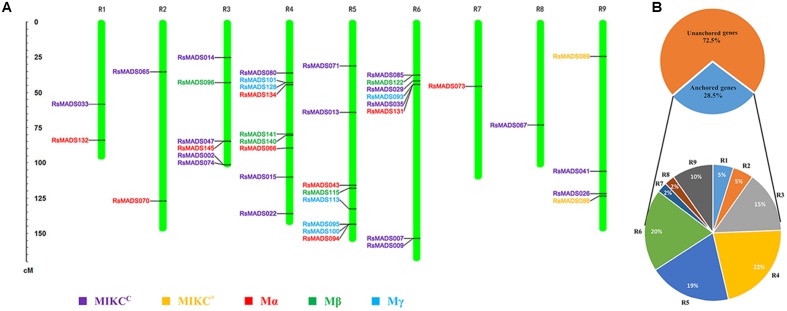
**Linkage group (LG) localization of *RsMADS* genes**. **(A)** Distribution of the *RsMADS* genes on nine radish LGs. **(B)** The percentage of *RsMADS* gene anchored onto each LG.

In total, 41 *RsMADS* genes (20 type I and 21 type II) accounting for 28.5% of the total MADS-box gene number were separately anchored onto the approximate location of linkage group (LG) R1-R9 (**Figure 3**; **Supplementary Table S5**). On the whole, the distribution of 41 *RsMADS* genes was relatively dispersed, but there were also some *RsMADS* gene cluster, for example, six genes clustered in the front of LG R2. Among the nine LGs, LG R4 contained the most *RsMADS* genes (9 members, ∼22%), while LG R7 and R8 presented the least member (1 member, ∼2%) (**Figure 3**). Moreover, there were two MIKC^∗^-type genes that were successfully anchored on the LG R9.

**FIGURE 4:**
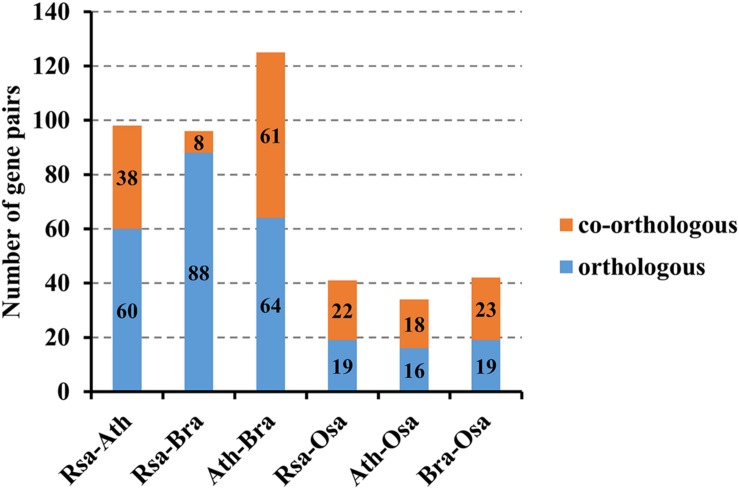
**The number of orthologous, co-orthologous gene pairs among radish (Rsa), *A. thaliana* (Ath), Chinese cabbage (Bra) and rice (Osa)**.

In the present study, orthologous and paralogous MADS-box genes between radish and other three plant species (Chinese cabbage, *A. thaliana* and rice) were comparatively analyzed by OrthoMCL Software. Among all the MADS-box genes, 60 orthologous and 38 co-orthologous gene pairs were found between radish and *A. thaliana.* Nevertheless, only 19 orthologous and 22 co-orthologous gene pairs were detected between radish and rice, and 16 orthologous and 18 co-orthologous gene pairs were found between *A. thaliana* and rice (**Figure 4**; **Supplementary Table S6**). Furthermore, a relational graph was used to visualize all the relationships among the orthologous, co-orthologous and paralogous MADS-box genes between radish and *A. thaliana* (**Supplementary Figure S3**). 29 and 50 *AtMADS* genes were determined to have no and only one orthologous gene in radish, respectively. While, 68 and 70 paralogous MADS-box gene pairs were detected in radish and *A. thaliana*, respectively (**Supplementary Figure S3**).

### Characterization of RsMADS Proteins, Conserved Motif Distribution and Intron–Exon Structure

To gain insight into the molecular characterization of 144 RsMADS proteins, their physical and chemical properties including molecular weights (MWs), theoretical isoelectric points (pI), instability index and aliphatic index were analyzed, and all RsMADS proteins were hydrophilic (**Supplementary Table S2**, **Supplementary Figure S4**). To analyze the features of RsMADS protein sequences, the 15 conserved motifs of 144 RsMADS proteins within the different groups were predicted by performing MEME motif search tool, and the LOGO of 15 amino acid motifs were generated (**Supplementary Figure S5**). Moreover, all of the RsMADS proteins contained motif 1 and motif 3, which indicated that this highly conserved domain was MADS domain. Nevertheless, motif 4 and motif 6 were present in most of the MIKC-type genes, and thus were predicted to be K-box domain. In addition, 15 motifs were submitted to the Pfam and SMART website for further identification, and provided strong evidences supporting our predictions (**Supplementary Figure S6**). Moreover, according to previous reports (Saha et al., 2015; Rameneni et al., 2014; Shu et al., 2013) and the conservative characteristics of motifs, motif 8 was predicted to represent the I domain, and motif 10 and motif 14 specified the C-terminal domain. It should be emphasized that type I group had more distinct motifs at their C-terminal regions except the MADS domain, which were more divergent than those in the type II group, and these motifs were identified as unknown by Pfam and SMART (**Supplementary Figure S6D**). Motif analysis showed that the majority of RsMADS proteins in the same subgroup shared similar motif distribution, suggesting that the proteins from the same subgroup probably had similar functions (Parenicová et al., 2003; Song et al., 2015).

Additionally, the intron–exon patterns were analyzed to investigate the structural diversity of *RsMADS* genes. Comparison of genomic DNA and cDNA showed that type I *RsMADS* genes had no or only one intron except *RsMADS133* containing five introns (**Supplementary Figure S6C**, **Supplementary Table S2**). Compared with type I, type II *RsMADS* genes had more complex structures. The intron numbers of type II *RsMADS* varied from 0 to 16 with an average of 5.6, and 60 (78.9%) members were consisted of at least five introns (**Supplementary Figure S6C**, **Supplementary Table S2**).

### Analysis of miRNAs Targeting *RsMADS* Genes

To have a better understanding of the function of MADS-box gene family in radish, a comprehensive miRNA library consisting of five miRNA libraries reported from our previous studies was used to determine miRNAs targeting *RsMADS* genes by psRNATarget program. Totally, 19 known miRNAs and six potential novel miRNAs (named Rsa-miR1-Rsa-miR6) belonging to 25 miRNA families were identified as putative miRNAs which could target 25 *RsMADS* target transcripts (**Supplementary Table S7**). The regulatory relationship between putative miRNAs and their targets were presented in **Supplementary Figure S7**. *RsMADS027* was the target transcript of miR8154, miR5293 and miR831-5p, *RsMADS084* was targeted by miR5174e-5p, Rsa-miR4 and Rsa-miR3, while four transcripts (*RsMADS087*, *RsMADS125*, *RsMADS138* and *RsMADS140*) were targeted by miR5021 (**Supplementary Figure S7**). It is worth noting that miR824 was predicted to target *RsMADS020* and *RsMADS044*, whose sequences showed high similarity with *AGL16* in *A. thaliana* (**Supplementary Table S2**).

### Differential Expression Analysis of *RsMADS* Genes

**FIGURE 5:**
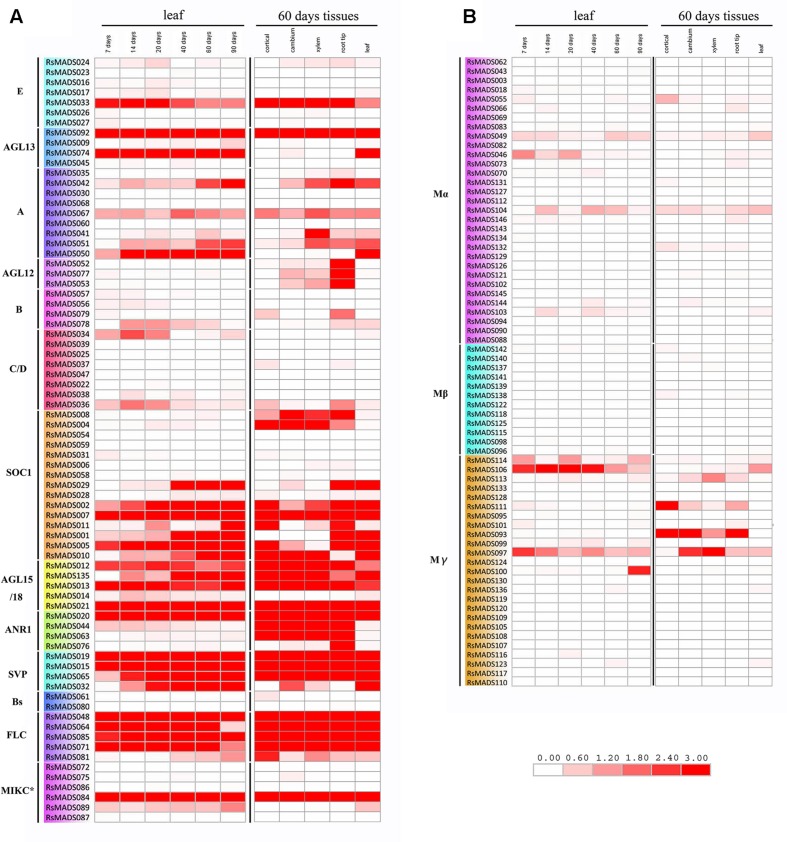
**Expression heat map of *RsMADS* genes in different stages and tissues. (A,B)** Represent Type II and Type I *RsMADS* expression profiles in six stages (7, 14, 20, 40, 60 and 90 DAS) and five tissues (cortical, cambium, xylem, root tip and leaf). The expression value was calculated by reads per kilobase per million reads (RPKM). The subgroup is marked on the left side of the gene list. The scale represents relative expression value.

To estimate the expression levels of *RsMADS* genes, RPKM of 144 *RsMADS* genes in leaves from seven different development stages and in five different tissues was obtained (Mitsui et al., 2015). The results showed that the transcript abundances of different *RsMADS* genes were extremely diverse in radish (**Figure 5**; **Supplementary Table S8**). On the whole, almost all Type I *RsMADS* genes either maintained a relatively low transcriptional level or had no expression in RNA-Seq libraries except *RsMADS093*, *RsMADS097*, *RsMADS106* and *RsMADS111* (**Figure 5B**). The expression of *RsMADS097* and *RsMADS106* were downregulated in leaves with the development of radish. *RsMADS093* and *RsMADS111* have high expression levels in roots but were hardly expressed in the leaves, indicating that they perhaps were root-specific and play a vital role in root development (**Figure 5B**).

Compared with Type I *RsMADS* genes, Type II genes showed a higher expression level both in the roots and leaves except subgroup B and C/D. With the growth of radish, expression levels of some genes increased gradually, including *RsMADS042*, *RsMADS050* (subgroup A); *RsMADS001*, *RsMADS002*, *RsMADS010*, *RsMADS029* (subgroup SOC1); *RsMADS135* (subgroup AGL15/18); *RsMADS032*, *RsMADS065* (subgroup SVP), while others decreased such as *RsMADS33* (subgroup E), *RsMADS13* (subgroup AGL15/18), *RsMADS44* (subgroup ANR1) and *RsMADS36* (subgroup C/D) (**Figure 5A**). In leaves and roots, the ABCDE model genes showed low transcript levels, while SOC1, AGL15/18, ANR1, SVP and FLC categories had high expression levels (**Figure 5A**). Interestingly, some genes exhibited tissue-specific expression (**Figure 5A**). For example, *RsMADS074* (subgroup AGL13), *RsMADS050* (subgroup A) and *RsMADS089* (subgroup MIKC^∗^) were specifically expressed in leaves, whereas some genes such as *RsMADS004*, *RsMADS008* (subgroup SOC1) and *RsMADS043*, *RsMADS044* (subgroup ANR1), were specifically expressed in roots. In addition, *RsMADS052*, *RsMADS053* and *RsMADS077* (subgroup AGL12) displayed a high expression level in root tips (**Figure 5A**).

### Expression Analysis of MIKC^C^ Genes by RT-qPCR

To further reveal the function of 12 subgroups of radish MIKC^C^ genes, the relative expression levels of genes in A, B, C/D, E, AGL6/13 and AGL12 subgroups were comprehensively investigated in various parts of floral organs (sepal, petal, stamen and carpel), and the genes in SOC1, AGL15/18, AGL16, SVP, Bs and FLC subgroups were validated under different GA concentrations, light length and vernalization time using RT-qPCR (**Supplementary Figure S8**).

**FIGURE 6:**
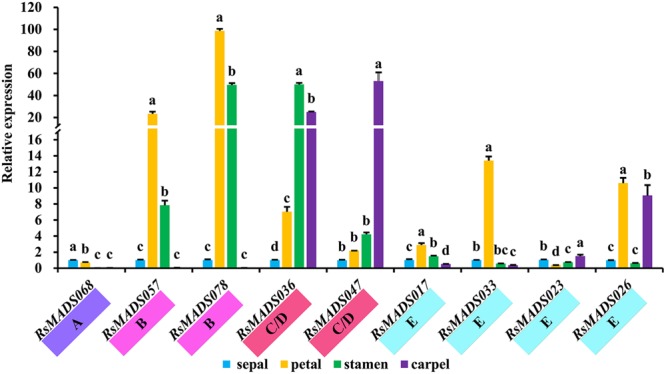
**The expression levels of representative *RsMADS* genes at different flower whorls including sepals, petals, stamens, carpels and ovules**. The subgroup is marked in different color under the gene name. Each bar shows the mean ± SE of the triplicate assay. The value with different letter indicates significant difference at *P* < 0.05 according to Duncan’s multiple range tests.

All *RsMADS* genes showed differential expression patterns in different parts of floral organs (**Supplementary Figure S8**). The orthologous *RsMADS* genes with *A. thaliana* ABCDE model genes were selected for further analysis. *RsMADS68* (*AP1*) exhibited high expression level in sepal and petal, as compared with that in stamen and carpel, whereas *RsMADS057* (*AP3*) and *RsMADS078* (*PI*) were significantly expressed in petal and stamen (**Figure 6**). More interestingly, *RsMADS036* (*AG*) tended to be expressed in stamen and carpel, while *RsMADS047* (*STK*) was significantly up-regulated only in carpel (**Figure 6**). Moreover, the expression patterns of E subgroup genes were more diverse. The expression levels of *RsMADS017* (*SEP1*) and *RsMADS023* (*SEP2*) were relatively steady in the four tissues, whereas *RsMADS033* (*SEP3*) and *RsMADS026* (*SEP4*) maintained relatively high expression levels in one (petal) and two (petal and carpel) specific tissues, respectively (**Figure 6**).

**FIGURE 7:**
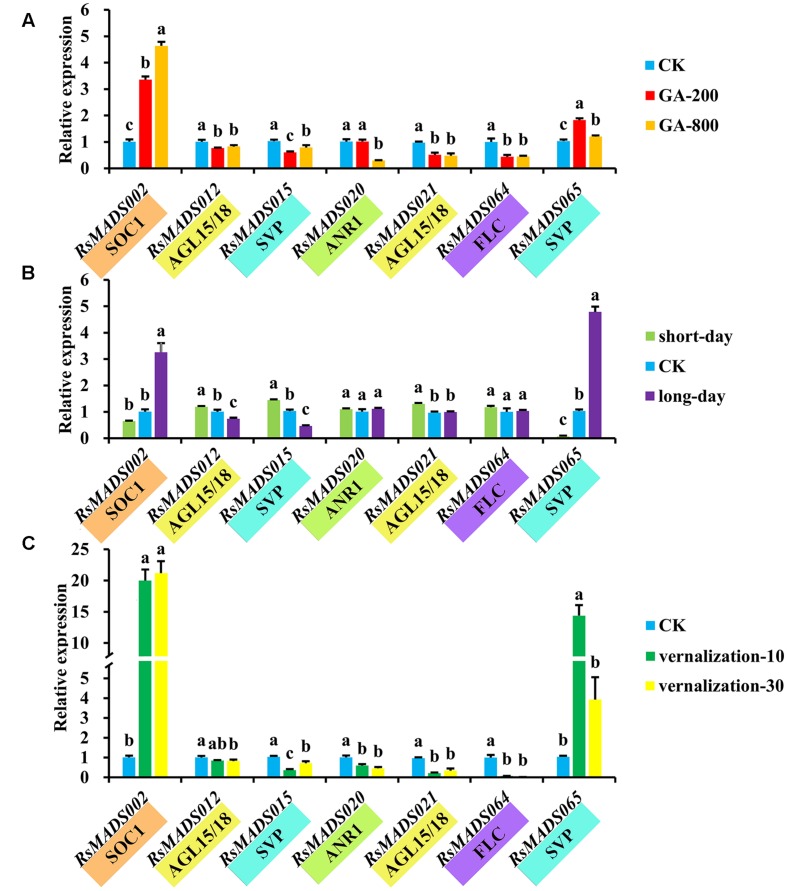
**The expression levels of representative *RsMADS* genes under GA **(A)**, photoperiod **(B)** and vernalization **(C)** treatments**. The subgroup is marked in different colors under the gene name. Each bar shows the mean ± SE of the triplicate assay. The value with different letter indicates significant difference at *P* < 0.05 according to Duncan’s multiple range tests.

Additionally, it is apparent that three different treatments (GA, light and vernalization) resulted in a wide variety of expression profiles among *RsMADS* genes (**Supplementary Figure S8**). The orthologs of seven representative genes including *RsMADS002*, *RsMADS012*, *RsMADS020*, *RsMADS021*, *RsMADS015*, *RsMADS064* and *RsMADS065* were reported to be crucial in flowering control in *A. thaliana*. The results showed that *RsMADS002* (*SOC1*) and *RsMADS065* (*AGL24*) were up-regulated under different concentrations of GA treatments, but *RsMADS015* (*SVP*), *RsMADS020* (*AGL16*), *RsMADS021* (*AGL15*) and *RsMADS064* (*FLC*) were obviously down-regulated (**Figure 7A**). *RsMADS015* (*SVP*) were down-regulated following the decrease of light lengths, whereas the transcript accumulation of *RsMADS020* (*AGL16*) and *RsMADS065* (*AGL24*) were relative lower at short-day (SD) and peaked at long-day (LD) (**Figure 7B**). Intriguingly, most members showed strong sensitivity toward vernalization treatment. Along with prolonging vernalization time, *RsMADS002* (*SOC1*) and *RsMADS065* (*AGL24*) were evidently induced, by contrast, the other five genes were inhibited inordinately (**Figure 7C**).

### The MADS-Box Gene-Mediated Regulation Associated with Flowering and Floral Organogenesis

In the present study, 16 critical *RsMADS* genes including *RsMADS002* (*SOC1*), *RsMADS012* (*AGL18*), *RsMADS015* (*SVP*), *RsMADS020* (*AGL16*), *RsMADS021* (*AGL15*), *RsMADS064* (*FLC*), *RsMADS065* (*AGL24*), *RsMADS68* (*AP1*), *RsMADS057* (*AP3*), *RsMADS078* (*PI*), *RsMADS036* (*AG*), *RsMADS047* (*STK*), *RsMADS017* (*SEP1*), *RsMADS033* (*SEP3*), *RsMADS023* (*SEP2*) and *RsMADS026* (*SEP4*) were identified to be involved radish flowering and floral organ formation. According to the reported *A. thaliana* and radish flowering and floral organogenesis regulatory network (Posé et al., 2012; Sun et al., 2013; Zhou et al., 2013; Khan et al., 2014; Nie et al., 2016), some critical floral pathway integrator genes, such as *FLC*, *SVP*, *AGL16*, *SOC1*, *AGL24* and *AGL19*, were considered to respond to environmental and endogenous factors directly or indirectly through interacting with other genes, and then these genes further regulate the expression of downstream floral organ identity genes including *AP1*, *AG*, *AP3* and *SEP3*.

## Discussion

Higher plants routinely go through various phase transitions from germination to death mainly including juvenile phase, vegetative growth and reproductive development. The vegetative phase change is essential for plants in response to environmental and endogenous factors, so as to complete their life cycle and achieve reproduction successfully (Khan et al., 2014). Flowering, as a symbol of plants into the reproductive growth phase, is determined by a complex gene interaction which is composed of a crowd of flowering and organogenesis related genes including most of MADS-box family genes (Smaczniak et al., 2012). In recent years, the bioinformatics analysis of gene families in different species facilitated the identification of various gene families with the completion of the genome sequencing. MADS-box gene families were identified and analyzed at a genome-wide scale in a series of plant species such as *A. thaliana* (Parenicová et al., 2003), rice (Arora et al., 2007), Chinese cabbage (Duan et al., 2015), cucumber (Hu and Liu, 2012), soybean (Fan et al., 2013) and maize (Zhao et al., 2011). However, it is still deficient for the genome-wide identification and analysis of MADS-box genes in radish. In the present study, a comprehensive analysis of MADS-box genes was performed and a total of 144 MADS-box family members were identified from the whole radish genome.

### Overview of MADS-Box Gene Family in Radish

In the current study, apart from 18 species reported previously, MADS-box gene families from other 10 species were firstly identified (**Supplementary Tables S3** and **S4**). As previously observed, it could be suggested that the number of MADS-box genes in Angiospermae was obviously larger than that in other species belonging to Algae, Bryophyta and Lycophytes (**Figure 1A**), indicating that a great expansion of MADS-box gene family members occurred after the angiosperm evolution (Gramzow et al., 2014; Duan et al., 2015). Simultaneously, the analysis of phylogenetic relationships between radish and other plant species, especially *A. thaliana*, provided a solid foundation for better understanding the function of *RsMADS* genes (Wang et al., 2015; Duan et al., 2015). In addition, the intron–exon structure feature has a potential influence on alternative splicing of gene to a certain extent, and the function of the protein will be affected (Tian et al., 2015). For type II *RsMADS* genes with more complex structures, it could be inferred that type II genes had more variable and intricate function than type I genes, which was in accordance with previous results in *A. thaliana* (Parenicová et al., 2003), Chinese cabbage (Duan et al., 2015) and soybean (Fan et al., 2013).

Previous evidence has shown that known miR5227 and novel Rsa-miR4 played a role in the bolting and flowering process of radish by high-throughput sequencing technology (Nie et al., 2015). Therefore, their target genes *RsMADS115* (*AGL103*) and *RsMADS084* (*AGL30*) might be associated with regulation of bolting and flowering time in radish. Furthermore, previous observations confirmed that miR824-regulated *AGL16* inhibited flowering in *A. thaliana* (Hu et al., 2014). In this study, miR824 was identified to target *RsMADS020* and *RsMADS044* (*AGL16*), revealing that these target genes may contribute to flowering time repression in radish.

### Characterization of Critical *RsMADS* Genes in Flowering and Floral Organ Development

Biochemical and genetic studies have indicated that flowering and floral organogenesis can be modulated by MADS-box genes especially MIKC^C^ type in higher plants (Lee et al., 2013; Ó’Maoiléidigh et al., 2014). Meanwhile, a growing number of key MADS-box genes including *FLC*, *SOC1*, *SVP*, *AGL24*, *AGL16*, *AGL15* and *AGL18*, and ABCDE model genes involved in this process have been widely recognized (Ferrario et al., 2004; Khan et al., 2014).

Control of flowering time is an intricate genetic circuitry in response to various endogenous and exogenous cues (Wullschleger and Weston, 2012). In *A. thaliana*, molecular genetics and physiological studies revealed that five main pathways of vernalization, photoperiod gibberellin, autonomy and age controlled flowering time (Kim et al., 2009; Mutasa-Göttgens and Hedden, 2009; Srikanth and Schmid, 2011; Wang, 2014). In this study, expression profiles of seven representative genes were investigated and the results suggested that *RsMADS015* (*SVP*), *RsMADS020* (*AGL16*), *RsMADS021* (*AGL15*) and *RsMADS064* (*FLC*) might act as flowering repressor, while *RsMADS002* (*SOC1*) and *065* (*AGL24*) contributed to the flowering promotion in radish (**Figure 7**).

Flower meristem and floral identity had been explained perfectly by five kinds of genetic function genes (A-B-C-D-E), which were important in regulating different flower whorls from sepals to carpels (Ferrario et al., 2004; Li et al., 2015). In the present study, the regulatory relationships between ABCDE genes and floral organ development were analyzed in radish, and a schematic ABCDE model was proposed (**Supplementary Figure S9**). RNA-Seq and RT-qPCR analysis revealed the expression patterns of ABCDE model orthologous genes in different tissues and at different stages of flower development. These genes exhibited relatively low abundant transcripts in the leaf and root (**Figure 5A**) and a regular expression patterns in different flower whorls (**Figure 6**; **Supplementary Figure S8A**), which were consistent with previous studies (Su et al., 2013; Ó’Maoiléidigh et al., 2014; Xie et al., 2015), suggesting that ABCDE model genes worked in a combinatorial manner to regulate the floral morphogenesis in radish.

### The Roles of MADS-Box Genes in Flowering and Flower Formation in Radish

Flowering is a coherent and sophisticated development process (Nie et al., 2015). Flowering-related genes were affected by multiple flowering signals converging on the regulation of floral organ identity genes including *SEP3*, *AP1*, *AG* and *AP3*, leading to flower formation eventually (Posé et al., 2012; Sun et al., 2013; Zhou et al., 2013; Khan et al., 2014). Considerable reports have indicated that *FLC* and *SOC1* as floral pathway integrators which were regulated by numerous genes and flowering pathways, played important roles in the flowering process (Lee et al., 2007; Franks et al., 2015). Genetic studies showed that *FLC* could block the transcriptional activation of *SOC1* and required *SVP* and *FRI* to delay flowering strongly (Helliwell et al., 2006; Lee et al., 2007; Geraldo et al., 2009; Franks et al., 2015). *AGL16*, a target gene of miR824, can help to repress flowering time by interacting indirectly with *FLC* and directly with *SVP* in *A. thaliana* (Hu et al., 2014). In this study, RT-qPCR validation showed that *FLC*, *SVP* and *AGL16* orthologous genes were down-expressed with the increase of GA concentration, light length and vernalization time (**Figure 7**), indicating that they may be repressors of flowering in radish.

Moreover, two other critical MADS-box genes, *SOC1* and *AGL24*, could promote flowering by responding to GA signaling (Moon et al., 2003). Additionally, *AGL15* and *AGL18* acted as the floral repressors via controlling the regulation of *SOC1* and *FT*, and *agl15 agl18* mutations presented a quick increase in *SOC1* and *FT* levels, leading to early flowering (Adamczyk et al., 2007; Fernandez et al., 2014). In the current study, *AGL24*, *SOC1*, *AGL15*, and *AGL18* orthologous genes were identified in radish, and RT-qPCR profiling showed that *AGL24* and *SOC1* orthologous genes were up-regulated, while *AGL15* and *AGL18* orthologous genes were obviously down-regulated when treated with different flowering-induced factors (**Figure 7**). These results suggested that *AGL24* and *SOC1* promoted flowering, whereas *AGL15* and *AGL18* inhibited flowering in radish. Therefore, it could be suggested that MADS-box gene family play a major role in regulating flowering time and floral meristem identity in radish.

## Conclusion

In conclusion, a total of 144 genes encoding MADS-box TF including 68 type I and 76 type II genes were identified in the whole radish genome. Among them, 41 genes were localized on the nine linkage groups of radish. A comparative phylogenetic analysis of the MADS-box genes was carried out between radish and *A. thaliana* to classify the MADS-box proteins. Furthermore, identification of miRNAs targeting *RsMADS* transcripts shed a novel insight into the functions of *RsMADS* genes at transcriptional and post-transcriptional level. In addition, RT-qPCR analysis provided a better understanding of critical functions of candidate *RsMADS* genes involved in flowering and floral organ identity in radish. Taken together, in this study, radish MADS-box gene family was comprehensively characterized, which facilitated dissecting *RsMADS* gene-mediated molecular mechanism underlying flowering and floral organogenesis in radish.

## Author Contributions

All authors listed, have made substantial, direct and intellectual contribution to the work, and approved it for publication.

## Conflict of Interest Statement

The authors declare that the research was conducted in the absence of any commercial or financial relationships that could be construed as a potential conflict of interest.

## Footnotes

1 http://www.nodai-genome-d.org/

2 http://pfam.xfam.org/

3 http://www.ncbi.nlm.nih.gov/

4 http://smart.embl-heidelberg.de/

5 http://www.arabidopsis.org/

6 http://brassicadb.org/brad/

7 http://peppergenome.snu.ac.kr/

8 http://www.phytozome.net/

9 http://mapinspect.software.informer.com/

10 http://www.orthomcl.org/cgi-bin/OrthoMclWeb.cgi

11 http://web.expasy.org/protparam/

12 http://gsds.cbi.pku.edu.cn/

13 http://meme-suite.org/tools/meme

14 http://plantgrn.noble.org/psRNATarget/

15 http://bonsai.hgc.jp/~mdehoon/software/cluster/software.htm

16 http://jtreeview.sourceforge.net/

## Supplementary material

FIGURE S1 **The protein structure and multiple sequence alignment of the **(A)***RsMADS040* and **(B)***RsMADS091***.

FIGURE S2 **Phylogenetic tree of radish and other species MADS-box proteins. (A)** Phylogenetic tree of AtMADS proteins. **(B)** The phylogenetic tree of radish and *A. thaliana* MADS proteins. **(C)** Phylogenetic tree of radish, *A. thaliana* and Chinese cabbage MADS proteins. **(D)** Phylogenetic tree of RsMADS proteins.

FIGURE S3 **The networks of MADS-box genes in radish and *A. thaliana.*** This interrelation network was constructed using radish and *A. thaliana* orthologous, co-orthologous and paralogous gene pairs. **(A)** One orthologous gene pair between radish and *A. thaliana*. **(B)** A complex network of orthologous, co-orthologous and paralogous gene pairs. **(C)** Paralogous gene pairs in radish and *A. thaliana*, respectively. **(D)** Statistics of the number of orthologous, co-orthologous and paralogous gene pairs between radish and *A. thaliana.* Orthologous gene pairs are linked in red lines; co-orthologous gene pairs are linked in yellow lines; paralogous gene pairs in radish are linked in black lines; paralogous gene pairs in *A. thaliana* are linked in blue lines.

FIGURE S4 **The physical and chemical properties of RsMADS proteins. (A)** The distribution of putative isoelectric points and molecular weights. **(B)** The distribution of Grand Average of hydropathicity (GRAVY), instability index and aliphatic index.

FIGURE S5 **Sequence logos of MADS domains in radish**. The overall height of the stack indicates the level of sequence conservation. The height of residues within the stack indicates the relative frequency of each residue at that position.

FIGURE S6 **The analysis of RsMADS proteins and *RsMADS* genes structure. (A)** The classification of *RsMADS* genes. **(B)** Phylogenetic tree of RsMADS proteins. **(C)** Intron–exon structure distribution of 144 *RsMADS* genes. **(D)** Conserved motif distribution of 144 RsMADS proteins.

FIGURE S7 **Predicted targeted regulatory network between *RsMADS* genes and miRNAs**.

FIGURE S8 **Expression analysis of *RsMADS* at different flower whorls and different treatments**. Heat map representation and hierarchical clustering of *RsMADS* genes during sepals, petals, stamens, carpels and ovules **(A)**; and under vernalization, photoperiod and GA treatments **(B)**. The scale represents relative expression value. The subgroup is marked in different color on the right side of the gene list.

FIGURE S9 **Putative schematic ABCDE model of floral organ development in radish**.

TABLE S1 **The primer sequences used for RT-qPCR of actin and MIKC^C^ genes**.

TABLE S2 **The information of 144 RsMADS genes in radish**.

TABLE S3 **The Information of MADS-box genes of various species from the previous reports**.

TABLE S4 **The Information of MADS-box genes from different species identified in this study**.

TABLE S5 **The information of linkage group localization of *RsMADS* genes**.

TABLE S6 **The orthologous gene pairs and co-orthologous gene pairs in MADS-box proteins of radish, *Arabidopsis*, Chinese cabbage and rice, and the paralogous gene pairs among these species**.

TABLE S7 **The information of miRNAs targeting *RsMADS* genes identified from previous five libraries**.

TABLE S8 **The RPKM values of *RsMADS* genes**.
