# Supplementary figures and images for: Genome-Wide Characterization of the MADS-Box Gene Family in Radish (Raphanus sativus L.) and Assessment of Its Roles in Flowering and Floral Organogenesis
Source: Front Plant Sci. 2016 Sep 20;7:1390. doi: 10.3389/fpls.2016.01390 (PMC5028395; doi:10.3389/fpls.2016.01390)

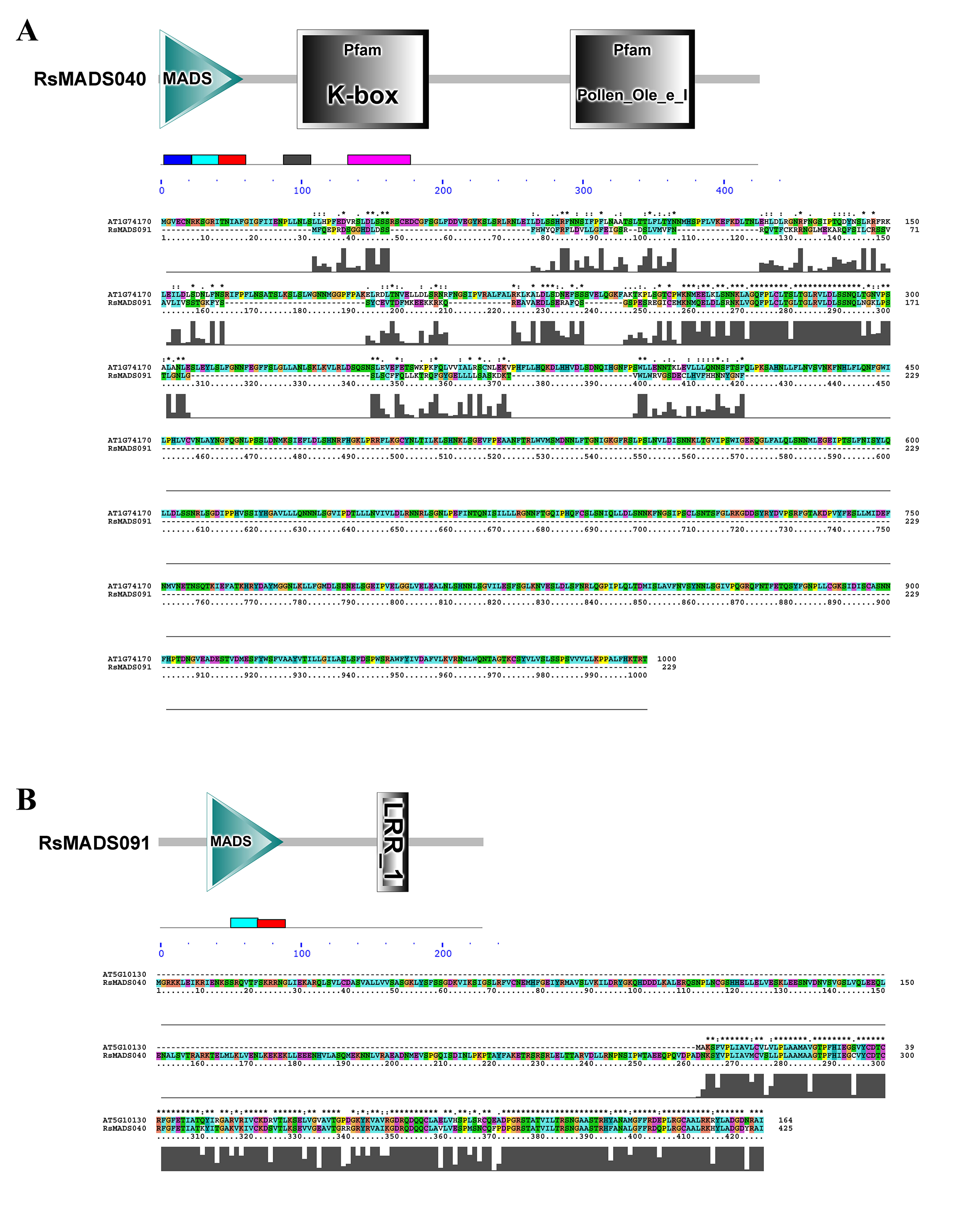

Supplement: FIGURE S1 — The protein structure and multiple sequence alignment of the (A)RsMADS040 and (B)RsMADS091. [file Image_1.TIF]

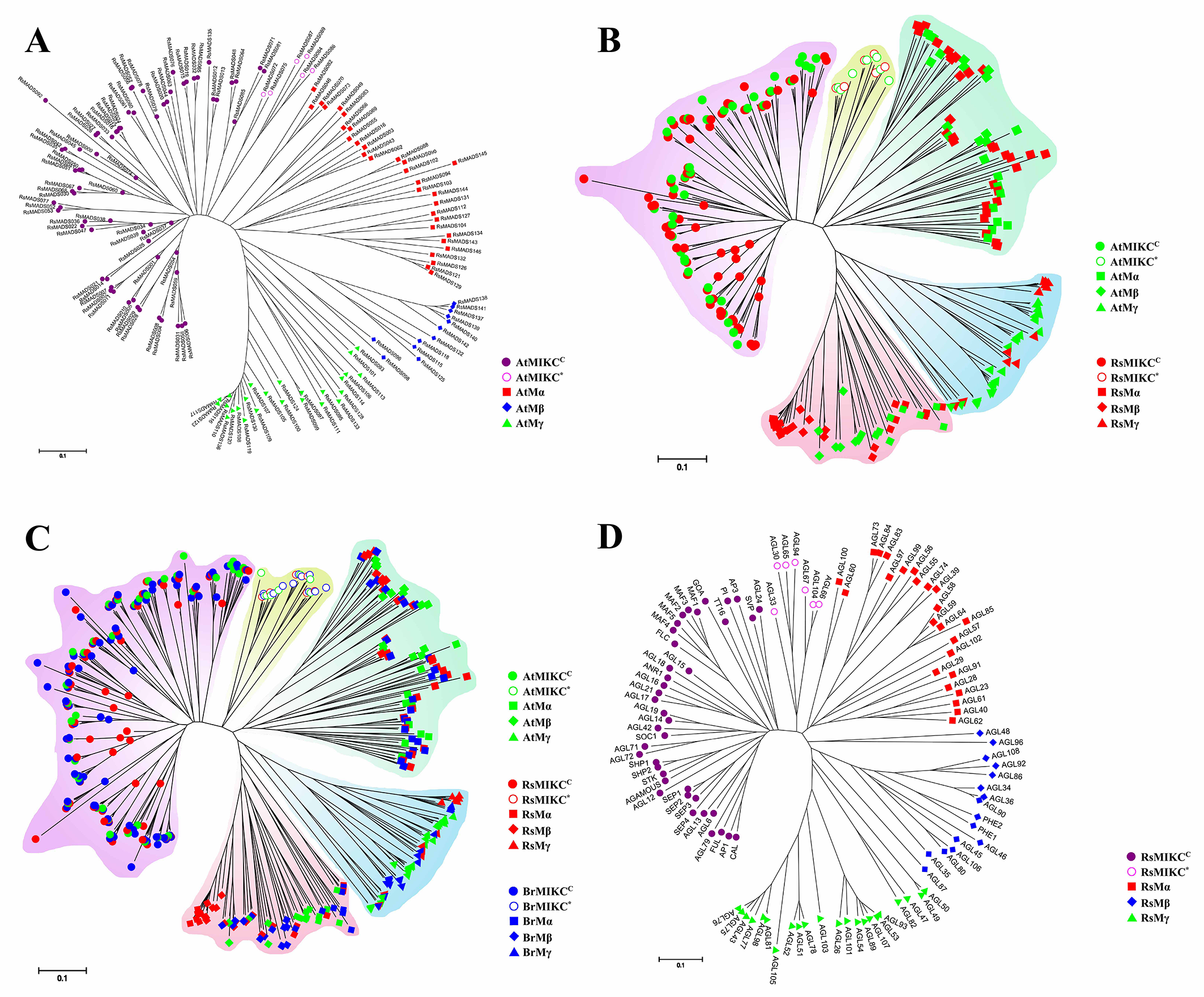

Supplement: FIGURE S2 — Phylogenetic tree of radish and other species MADS-box proteins. (A) Phylogenetic tree of AtMADS proteins. (B) The phylogenetic tree of radish and A. thaliana MADS proteins. (C) Phylogenetic tree of radish, A. thaliana and Chinese cabbage MADS proteins. (D) Phylogenetic tree of RsMADS proteins. [file Image_2.TIF]

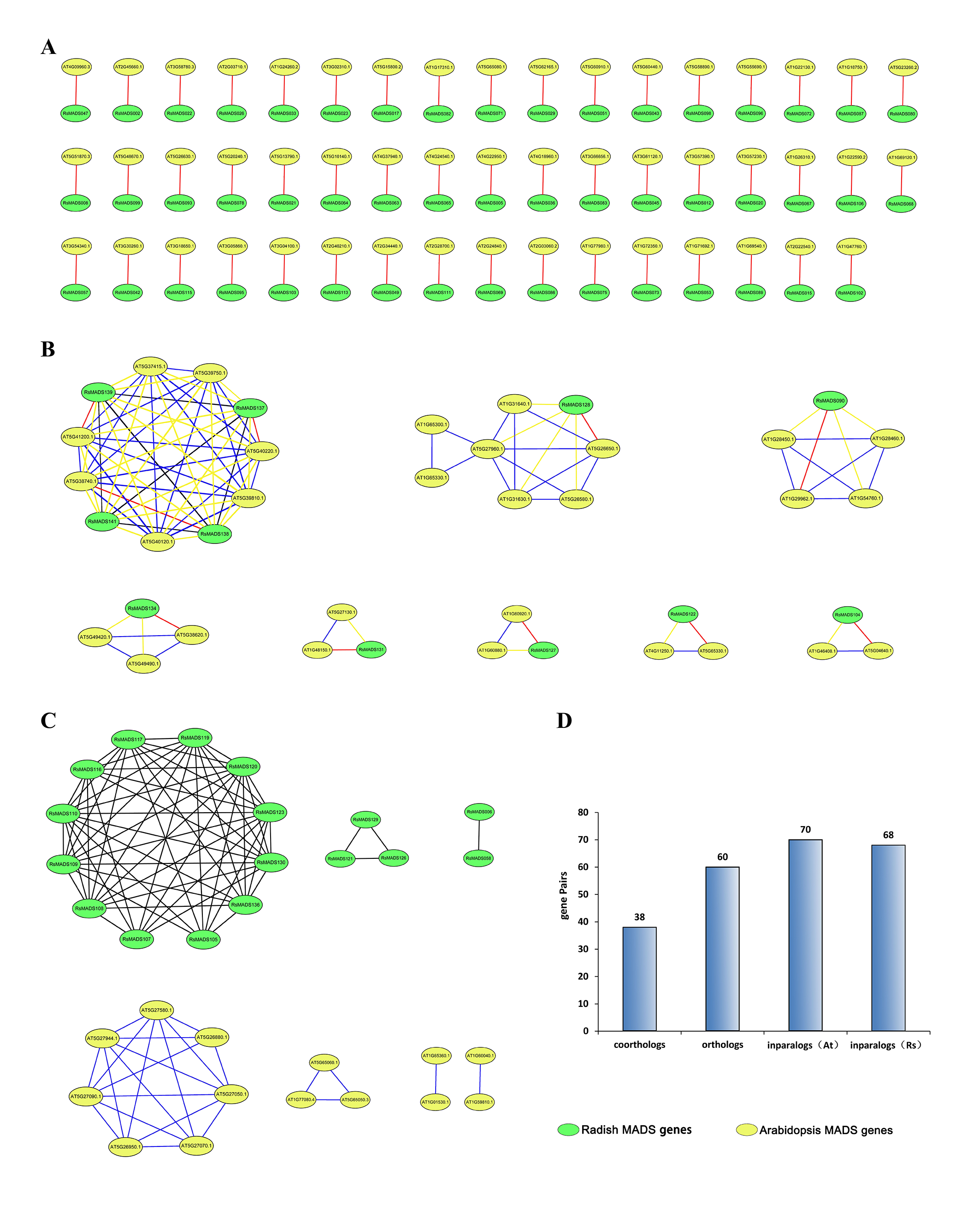

Supplement: FIGURE S3 — The networks of MADS-box genes in radish and A. thaliana. This interrelation network was constructed using radish and A. thaliana orthologous, co-orthologous and paralogous gene pairs. (A) One orthologous gene pair between radish and A. thaliana. (B) A complex network of orthologous, co-orthologous and paralogous gene pairs. (C) Paralogous gene pairs in radish and A. thaliana, respectively. (D) Statistics of the number of orthologous, co-orthologous and paralogous gene pairs between radish and A. thaliana. Orthologous gene pairs are linked in red lines; co-orthologous gene pairs are linked in yellow lines; paralogous gene pairs in radish are linked in black lines; paralogous gene pairs in A. thaliana are linked in blue lines. [file Image_3.TIF]

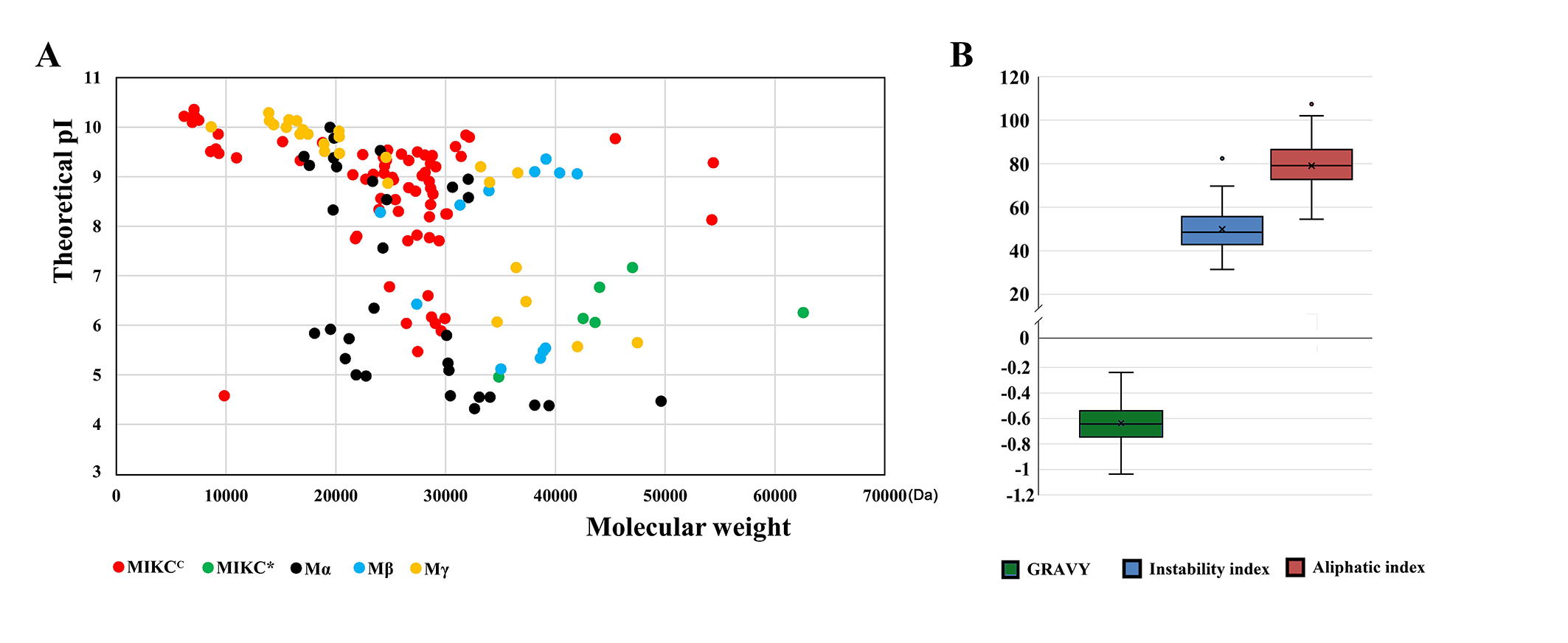

Supplement: FIGURE S4 — The physical and chemical properties of RsMADS proteins. (A) The distribution of putative isoelectric points and molecular weights. (B) The distribution of Grand Average of hydropathicity (GRAVY), instability index and aliphatic index. [file Image_4.TIF]

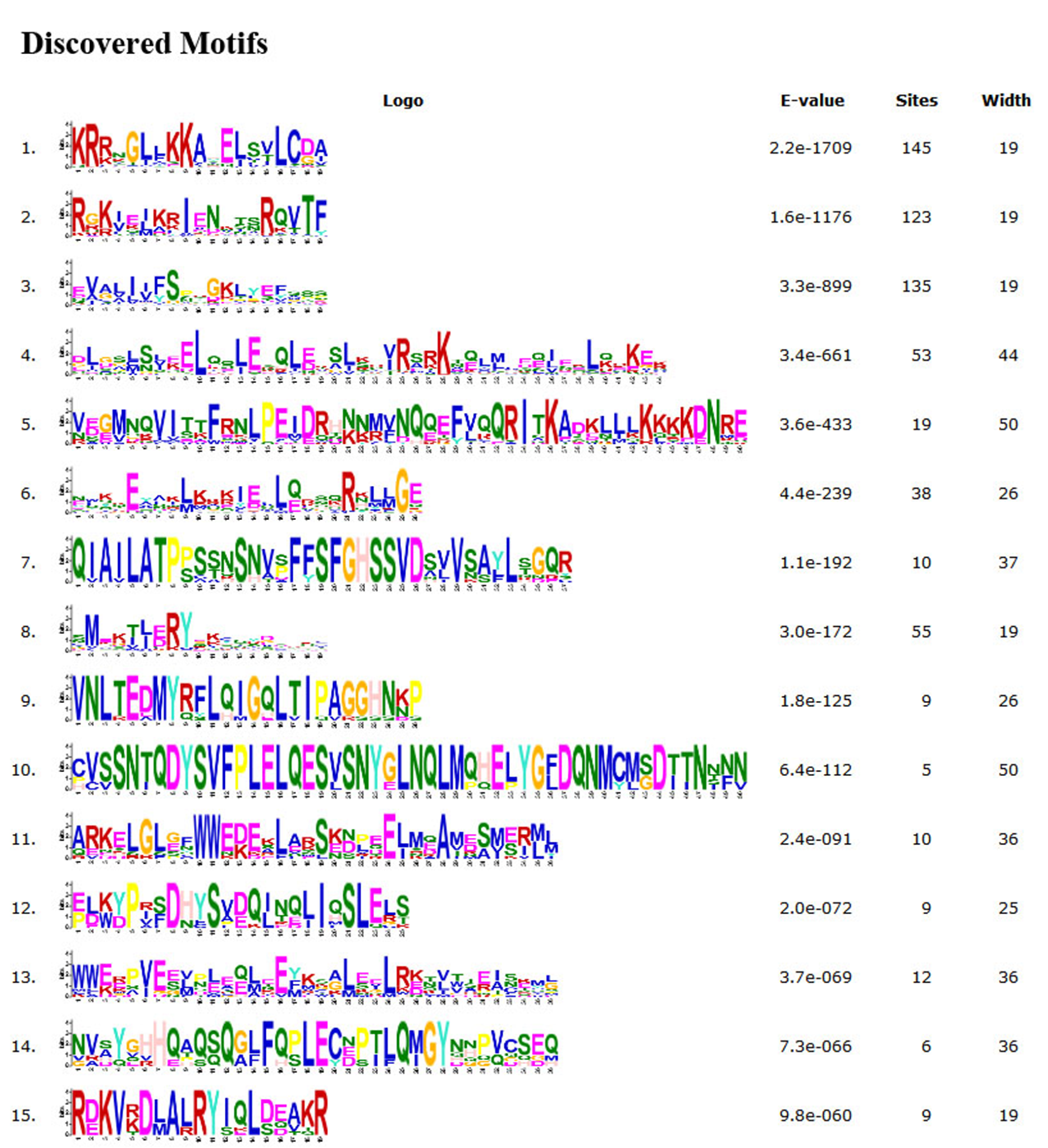

Supplement: FIGURE S5 — Sequence logos of MADS domains in radish. The overall height of the stack indicates the level of sequence conservation. The height of residues within the stack indicates the relative frequency of each residue at that position. [file Image_5.TIF]

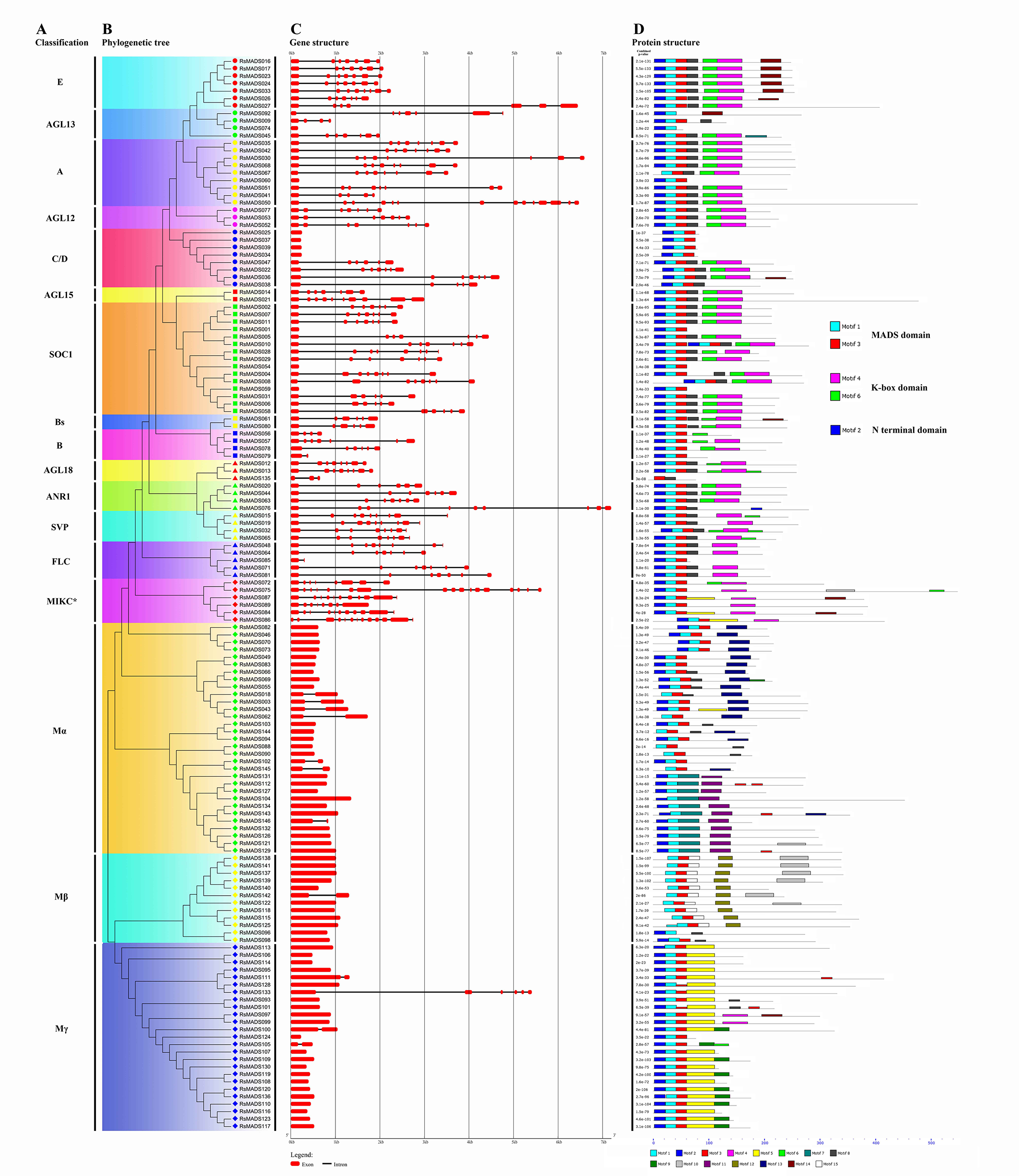

Supplement: FIGURE S6 — The analysis of RsMADS proteins and RsMADS genes structure. (A) The classification of RsMADS genes. (B) Phylogenetic tree of RsMADS proteins. (C) Intron–exon structure distribution of 144 RsMADS genes. (D) Conserved motif distribution of 144 RsMADS proteins. [file Image_6.TIF]

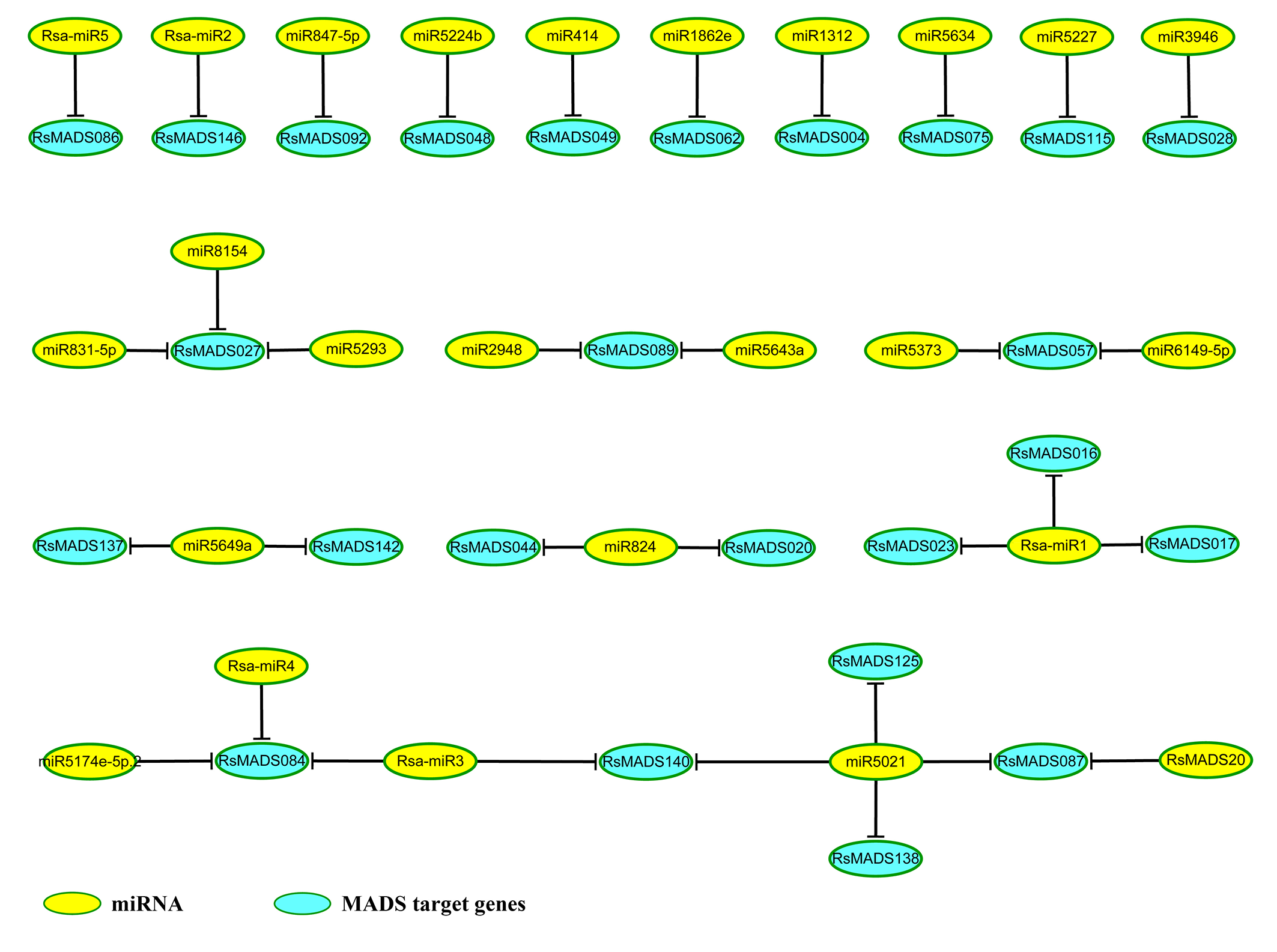

Supplement: FIGURE S7 — Predicted targeted regulatory network between RsMADS genes and miRNAs. [file Image_7.TIF]

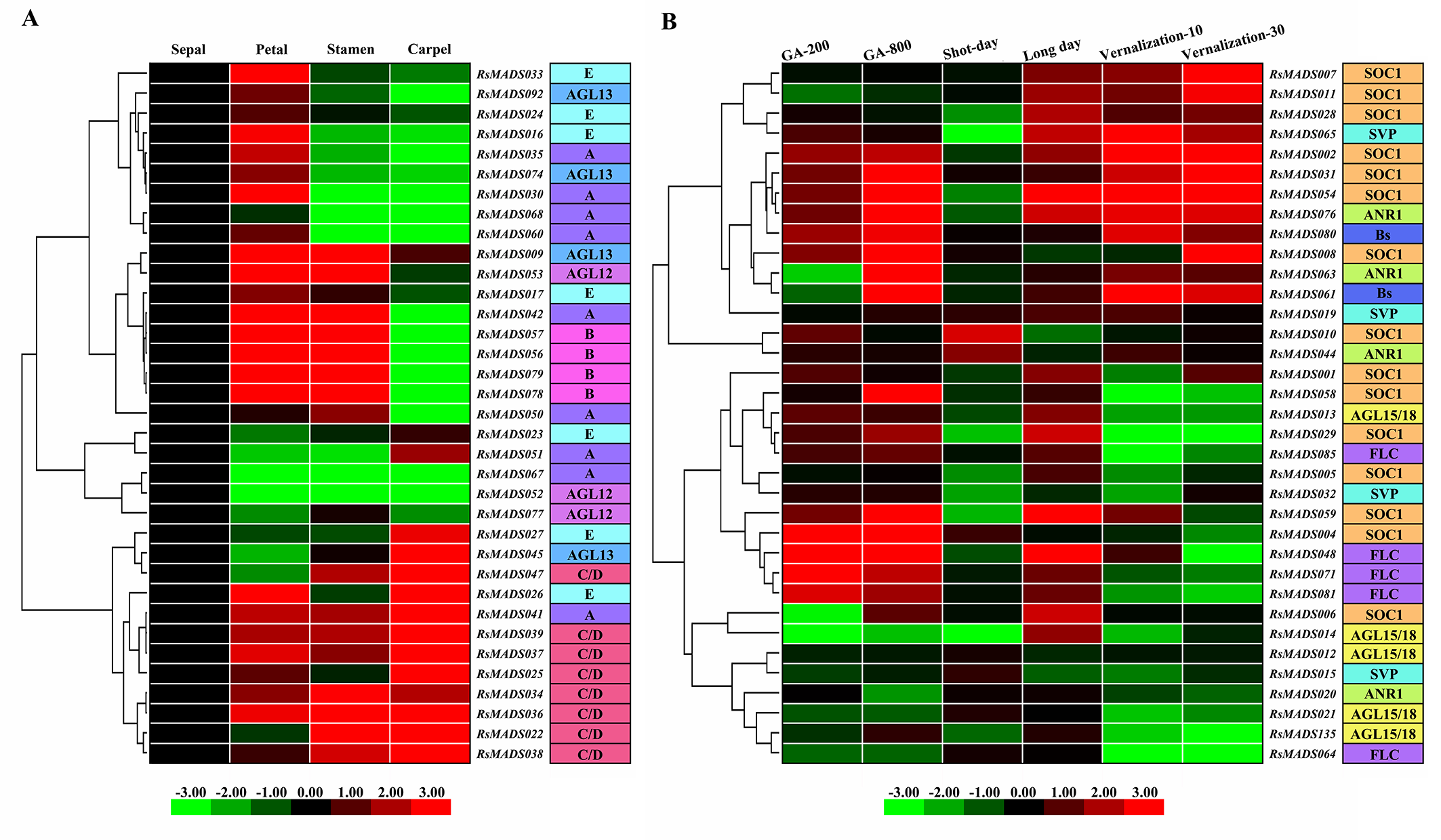

Supplement: FIGURE S8 — Expression analysis of RsMADS at different flower whorls and different treatments. Heat map representation and hierarchical clustering of RsMADS genes during sepals, petals, stamens, carpels and ovules (A); and under vernalization, photoperiod and GA treatments (B). The scale represents relative expression value. The subgroup is marked in different color on the right side of the gene list. [file Image_8.TIF]

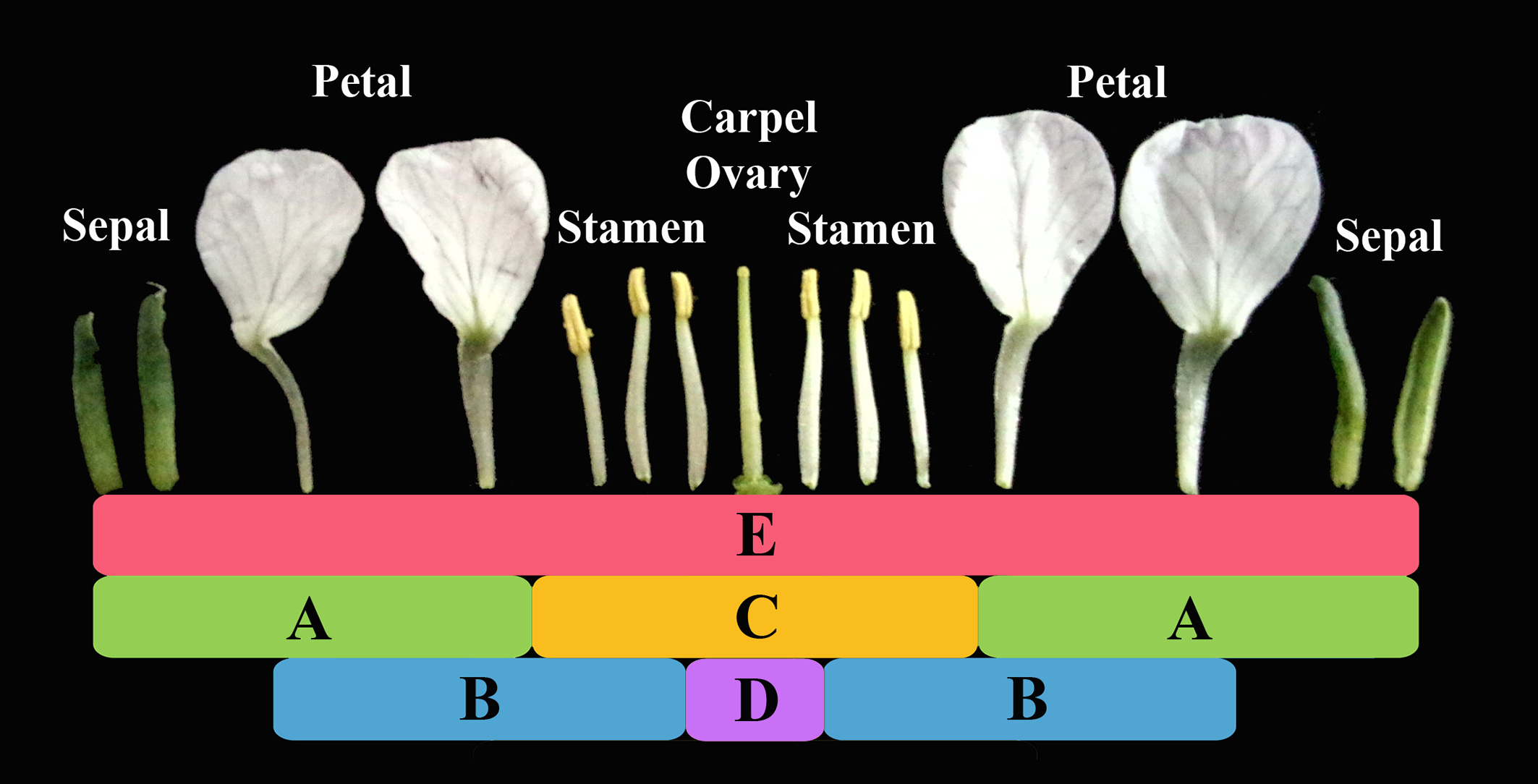

Supplement: FIGURE S9 — Putative schematic ABCDE model of floral organ development in radish. [file Image_9.TIF]
